# Perioperative anesthetic management for resection of giant retroperitoneal tumors

**DOI:** 10.3389/fonc.2026.1833254

**Published:** 2026-07-01

**Authors:** Xiaoxia Zhang, Han Wei, Miao He, Xiaoli Sun, Zhenyuan Wang, Mingying Li

**Affiliations:** Department of Anesthesiology, Beijing Chao-Yang Hospital, Capital Medical University, Beijing, China

**Keywords:** anesthetic management, case series, giant tumor, perioperative management, retroperitoneal tumor

## Abstract

**Objective:**

The aim of this study was to summarize perioperative anesthetic management in patients undergoing resection of giant retroperitoneal tumors and to provide a reference for clinical practice.

**Methods:**

This retrospective study analyzed data from 10 patients who underwent elective resection of giant retroperitoneal tumors (maximum diameter ≥ 15 cm) at a single institution between January 2018 and December 2023. Demographic characteristics, tumor features, preoperative preparations, intraoperative hemodynamic data, fluid and blood product administration, and postoperative complications were collected and analyzed. Descriptive statistical methods were applied.

**Results:**

The cohort included 1 male and 9 female patients, with median mean age of 56.5 year-old (range, 24–79 year-old). The median maximum tumor diameter was 33 cm (range, 22–50 cm). Preoperative multidisciplinary team consultations were conducted in 8 patients, and interventional vascular embolization was performed in 7 patients. All patients received general anesthesia with endotracheal intubation. Invasive arterial pressure monitoring was used in all patients, central venous pressure monitoring in 9, and FloTrac™/Vigileo monitoring in 6. The median operative duration was 260 minutes. The median intraoperative blood loss was 2,700 mL, and the median cumulative duration of hypotension was 32 minutes. Eight patients received blood transfusions (The median cumulative transfusion volume of red blood cells and plasma was 2,650 mL). Vasoactive agents were administered in all patients. Postoperative complications included nausea and vomiting (3 cases), atelectasis (2 cases), pleural effusion (2 cases), and surgical site infection (2 cases). No perioperative mortality occurred.

**Conclusion:**

The principal anesthetic challenges during resection of giant retroperitoneal tumors were hemodynamic instability and massive hemorrhage. Comprehensive preoperative preparation, advanced hemodynamic monitoring, proactive management of intravascular volume, and adequate availability of blood products may be critical components in improving patient outcomes.

## 1 Introduction

Retroperitoneal tumors are neoplasms that arise in the retroperitoneal space, including the presacral and pelvic floor regions. Based on biological behavior, these tumors are classified as benign, malignant, or borderline, with malignant tumors representing the most common type (1, 2). Tumors with a maximum diameter ≥ 15 cm or a weight ≥ 2 kg are defined as giant masses (3). Giant retroperitoneal masses are uncommon in clinical practice, and their surgical resection is associated with substantial operative risk. The retroperitoneal space has a complex anatomical configuration and is located adjacent to major vascular structures, including the abdominal aorta and inferior vena cava, as well as critical nerves and abdominal organs. Large masses may exert significant compressive effects on these surrounding structures, resulting in complex pathophysiological changes. These factors create multiple challenges for perioperative anesthetic management, including hemodynamic instability, an increased risk of massive hemorrhage, and vascular compression (4).

The existing literature primarily comprises case reports, and comprehensive summaries of anesthetic management for these procedures remain limited. A retrospective analysis was conducted of perioperative anesthetic management data from 10 patients who underwent elective resection of giant retroperitoneal tumors at a single institution between January 2018 and December 2023. The objective was to summarize management strategies and provide a reference for clinical practice.

## 2 Data and methods

### 2.1 General data

Ethical approval was obtained from the hospital ethics committee (2025-Sci-1037), and the study was conducted in accordance with the ethical principles of the Declaration of Helsinki. All patient data were anonymized. Data from 10 patients who underwent elective resection of giant retroperitoneal tumors at a single institution between January 2018 and December 2023 were included.

The inclusion criteria were as follows: age ≥ 18 years; presence of a retroperitoneal tumor with a maximum diameter ≥ 15 cm or weight ≥ 2 kg; and availability of complete perioperative anesthetic records.

The exclusion criteria were as follows: emergency surgery; American Society of Anesthesiologists (ASA) physical status classification ≥ IV; pregnancy; or incomplete medical records.

### 2.2 Observation indicators

The following data were collected: demographic characteristics; preoperative comorbidities; imaging findings, including tumor location, tumor diameter, tumor multiplicity, presence of metastasis, and involvement of major abdominal vessels; and preoperative preparations, including multidisciplinary team (MDT) consultation, interventional vascular embolization of the tumors, and placement of a ureteral double-J (D-J) stent. Additionally, operative duration; intraoperative fluid input and output, including total infusion volume; intraoperative hemodynamic status and related management measures, including duration of hypotension, lowest recorded blood pressure values, and use of vasoactive agents; intraoperative adverse events; and postoperative complications were documented.

The most widely accepted clinical definition of intraoperative hypotension has been described as a reduction in blood pressure of > 20% from baseline, accompanied by an invasive systolic arterial pressure (SAP) ≤ 100 mmHg and a mean arterial pressure (MAP) ≤ 65 mmHg (5). In this study, intraoperative hypotension was defined as a systolic arterial pressure (SAP) ≤90 mmHg or a mean arterial pressure (MAP) <65 mmHg accompanied by a reduction in SAP exceeding 35% from baseline. Cumulative duration (in minutes) was used as the primary outcome measure.

### 2.3 Statistical analysis

Given that this study was designed as a case series with a small sample size (*n* = 10), descriptive statistical methods were primarily applied. As this was a case series with a small sample size (n = 10), and small-sample data frequently deviate from normality, all continuous variables were reported as medians (interquartile ranges), and no normality tests were conducted. All statistical analyses were performed using SPSS software (version 27.0). Inferential statistical analyses were not performed due to the limited sample size.

## 3 Case presentation

### 3.1 Basic characteristics and preoperative status of the patients

Among the 10 patients who met the inclusion criteria, 1 was male and 9 of were female. The median age was 56.5 (39.0, 72.0) years, and the median body mass index was 23.27 (20.68, 27.18) kg/m². Two patients were classified as ASA physical status II, and eight were classified as ASA physical status III. The observed comorbidities included anemia (*n* = 7), hypoproteinemia (*n* = 7), hypoxemia (*n* = 6), hypertension (*n* = 5), pulmonary dysfunction (defined as moderate-to-severe restrictive ventilatory impairment or inability to complete pulmonary function testing; *n* = 5), and coagulation disorders (*n* = 4). Cardiac dysfunction, renal dysfunction, adrenal cortical insufficiency, and hypothyroidism were each identified in one patient.

### 3.2 Characteristics of the tumors

Among the 10 patients, tumors originated in the retroperitoneal space in 7 patients, in the pelvis with invasion into the abdominal cavity and retroperitoneal space in 2 patients, and in the abdominal cavity with invasion into the pelvis and retroperitoneal space in 1 patient. The maximum tumor diameter ranged from 22 to 50 cm, with a median of 33 cm (range, 22–50 cm). Tumor diameter was ≥ 30 cm in 7 patients and ≥ 40 cm in 3 patients. Multifocal tumors were observed in 7 patients, and pulmonary metastasis was identified in 1 patient. The largest tumor weighed 16 kg. The pathological subtypes were as follows: dedifferentiated liposarcoma (*n* = 2), well-differentiated liposarcoma (*n* = 1), desmoid-type fibromatosis (*n* = 2), cellular leiomyoma (intravenous leiomyomatosis, *n* = 1), adult-type granulosa cell tumor of the ovary (*n* = 1), pleomorphic leiomyosarcoma (*n* = 1), gastrointestinal stromal tumor (high-risk category, n = 1), and fibrosarcoma (*n* = 1). Based on biological behavior, 7 tumors were malignant, 2 were borderline, and 1 was benign. In one patient with intravenous leiomyomatosis, tumor extension occurred along the inferior vena cava into the right atrium.

### 3.3 Preoperative preparation

Among the 10 patients, MDT consultation was conducted in 8 patients. Preoperative interventional vascular embolization of the tumors was performed in 7 patients, and preoperative placement of occlusive balloons in the abdominal aorta and iliac arteries was carried out in 1 patient. Preoperative placement of ureteral D-J stents was performed in 5 patients.

### 3.4 Anesthesia management

#### 3.4.1 Anesthesia administration

General anesthesia with rapid-sequence induction and endotracheal intubation was administered in all 10 patients. The induction regimen consisted of sufentanil 0.3–0.5 μg/kg, etomidate 0.1–0.2 mg/kg, and rocuronium bromide 0.6 mg/kg or cisatracurium besylate 0.1–0.2 mg/kg. Endotracheal intubation was facilitated using a video laryngoscope, and mechanical ventilation was provided using a Mindray anesthesia machine. Volume-controlled ventilation was applied with a fraction of inspired oxygen of 60%, tidal volume of 8–10 mL/kg, respiratory rate of 12–16 breaths per minute, and an inspiratory-to-expiratory ratio of 1:2. Ventilatory parameters were adjusted intraoperatively according to end-tidal carbon dioxide (PetCO_2_) levels to maintain PetCO_2_ between 35 and 45 mmHg. Anesthesia was maintained using a combined intravenous–inhalational technique. Sevoflurane at 1% was administered by inhalation, and continuous intravenous infusions of remifentanil at 0.2–0.5 μg/kg/min and propofol at 4–5 mg/kg/h were provided. Rocuronium bromide or cisatracurium besylate was administered intermittently as bolus doses to maintain bispectral index values between 40 and 60. For perioperative analgesia, bilateral ultrasound-guided transversus abdominis plane (TAP) blocks were performed in 4 patients, with 20 mL of 0.25% ropivacaine administered on each side.

#### 3.4.2 Intraoperative monitoring

Standard monitoring, including electrocardiography, non-invasive blood pressure measurement, pulse oximetry, and end-tidal carbon dioxide, was used in all patients. Advanced monitoring was implemented as follows: invasive continuous arterial pressure monitoring was established in all patients, with the radial artery serving as the primary site of catheterization. Central venous pressure monitoring was conducted in 9 patients, primarily via catheterization of the right internal jugular vein. FloTrac hemodynamic monitoring was used in 6 patients. Dynamic arterial blood gas analysis was performed in all patients, with a median of 4 analyses per patient. Key assessment time points included after anesthesia induction, during tumor manipulation, after tumor resection, and following reperfusion.

#### 3.4.3 Intraoperative management

The median number of established peripheral or central intravenous access routes during surgery was 3. A goal-directed fluid therapy (GDFT) strategy was used. Infusion rates and fluid types were adjusted according to hemodynamic monitoring parameters, including invasive arterial pressure, central venous pressure, cardiac index, stroke volume variation, and urine output. Vasoactive agents were administered as clinically indicated to maintain perioperative hemodynamic stability. Among the 10 patients, the median lowest intraoperative systolic blood pressure was 70 mmHg (range, 45–85 mmHg), and the median cumulative duration of hypotension was 32 minutes (range, 11–62 minutes). The median crystalloid infusion volume was 3,825 mL (range, 3,100–6,425 mL), and the median colloid infusion volume was 750 mL (range, 500–1,000 mL). Blood product transfusion was administered in 8 of the 10 patients. The median red blood cell (RBC) transfusion volume was 1,450 mL (range, 300–2,900 mL), and the median plasma transfusion volume was 1,100 mL (range, 150–2,000 mL). The median cumulative transfusion volume of red blood cells and plasma was 2,650 mL (range, 600-5,050 mL).

Platelet transfusion was conducted in 1 patient, and cryoprecipitate was not administered. Fibrinogen and prothrombin complex concentrate were administered in 2 patients. Vasoactive agents were administered in all 10 patients, including norepinephrine (*n* = 10), dopamine (*n* = 5), ephedrine (*n* = 2), and epinephrine (*n* = 1). These agents were primarily used to manage hypotension following anesthesia induction, hemodynamic fluctuations during tumor manipulation and resection, and to provide blood pressure support after resuscitation.

### 3.5 Perioperative outcomes and complications

The duration of surgery ranged from 145 to 440 minutes, with a median duration of 260 minutes. Intraoperative blood loss ranged from 200 to 7,500 mL, with a median of 2,700 mL. Massive hemorrhage (≥ 1,000 mL) occurred in 8 patients, and severe hemorrhage (≥ 5,000 mL) occurred in 3 patients. Complete tumor resection was achieved in 6 patients, whereas partial or near-total resection was performed in 4 patients. Immediate extubation following surgery was achieved in 3 patients. Postoperative mechanical ventilation was required in 7 patients, and admission to the intensive care unit (ICU) was required in 8 patients. The duration of ICU stay ranged from 0 to 12 days, with a median of 3.5 days.

Among the 10 patients, perioperative complications were observed in 7 patients. Anesthesia-related complications included postoperative nausea and vomiting in 3 patients; all cases were classified as mild to moderate and resolved following antiemetic therapy. No instances of respiratory depression or delayed emergence from anesthesia were observed. Pulmonary complications included bilateral lower lobe atelectasis in 2 patients and pleural effusion in 2 patients. Cardiovascular complications consisted of 1 case of acute myocardial injury and 1 case of cardiac dysfunction. Additional complications included 1 case of hemorrhagic shock, which was stabilized after massive transfusion and vasoactive support; as well as 2 cases of surgical site infection and 1 case of deep vein thrombosis. No perioperative mortality occurred. Perioperative outcomes are summarized in **Table 1**.

**Table 1:** Patient status during the perioperative period (*n* = 10).

Data Category	Indicator	Value
Preoperative comorbidity (Case)	Anemia	7
	Hypoproteinemia	7
	Hypoxemia	6
	Hypertension	5
	Pulmonary dysfunction.	5
	Coagulation disorder.	4
Preoperative imaging (Case)	The tumor is in the retroperitoneal space.	7
	The tumor is in the abdominal cavity, pelvic cavity, and retroperitoneal space.	3
	Diameter of tumor ≥ 30cm	7
	Diameter of tumor ≥ 40cm	3
	Multifocal tumor	7
	Metastasis	1
Preoperative preparation (Case)	multidisciplinary team (MDT) consultation	8
	Vascular embolization of the tumors	7
Duration of operation(min)	260(184,323)	
Intraoperative management (Case)	Invasive arterial pressure monitoring.	10
	Central venous pressure monitoring.	9
	Flotrac monitoring	6
	Lowest systolic pressure during operation (mmHg)	70(45,85)
	Duration of low blood pressure (min)	32(11,62)
	Blood loss ≥ 1,000ml (case)	8
	Blood loss ≥ 4,000ml (case)	3
	Volume of crystalloid infusion (ml)	3825(3100,6425)
	Volume of colloid infusion (ml)	750(500,1000)
	Volume of red blood cell transfusion (ml)	1450(300,2900)
	Volume of plasma transfusion (ml)	1100(150,2000)
	Use of vasoactive agents (Case)	10
Perioperative outcome	Complete removal of the tumor (Case)	6
	Partial removal of the tumor (Case)	4
	Removal of the tube immediately after the operation (Case)	3
	Transport the patient with the tube to the ICU (Case)	7

### 3.6 Typical case

Case 1: A 43-year-old female patient classified as ASA physical status II was admitted because of a giant abdominopelvic mass with a three-year history. Preoperative computed tomography (CT) demonstrated a giant mass involving the abdominopelvic cavity and retroperitoneal space, measuring up to 28 cm in maximum diameter and closely adherent to the inferior vena cava and abdominal aorta. The pathological diagnosis was cellular leiomyoma (intravenous leiomyomatosis). Preoperatively, an MDT consultation was conducted to establish an individualized anesthetic management plan. In view of the diagnosis of intravenous leiomyomatosis and the anticipated risk of substantial intraoperative hemorrhage during tumor resection, preoperative vascular embolization was performed on two occasions. On the day of surgery, occlusive balloons were placed in the abdominal aorta and iliac arteries to achieve temporary major vessel occlusion to reduce intraoperative blood loss.

Rapid-sequence induction of anesthesia was conducted. Invasive arterial pressure monitoring was established via the left radial artery, continuous cardiac output monitoring was implemented using the FloTrac system through a peripheral arterial line, and central venous pressure monitoring was established via the right internal jugular vein. Bilateral ultrasound-guided TAP blocks were performed for perioperative analgesia, with 20 mL of 0.25% ropivacaine administered on each side. Intraoperative monitoring demonstrated significant hemodynamic fluctuations during tumor resection. Invasive arterial pressure decreased to 59/31 mmHg, heart rate increased to approximately 100 beats per minute, and central venous pressure decreased to 2 cm H_2_O. Immediate pressurized blood transfusion and rapid volume resuscitation were initiated. Continuous infusion of norepinephrine at 0.1–0.4 μg/kg/min was administered, together with intermittent bolus doses of ephedrine and dopamine, resulting in gradual hemodynamic stabilization.

During tumor excision, despite intermittent balloon occlusion of the abdominal aorta and iliac arteries, the estimated blood loss reached approximately 5,600 mL. Transfusion included packed RBCs (4,600 mL) and plasma (1,800 mL). The total operative duration was 360 minutes. Postoperatively, the patient remained intubated and was transferred directly to the ICU. Extubation was performed 36 hours after surgery, and the ICU stay lasted 7 days. Postoperative recovery was favorable, and no severe complications were observed.

Case 2: A 56-year-old male patient classified as ASA physical status III was admitted following the identification of a retroperitoneal mass one year earlier. Preoperative evaluation demonstrated dilated cardiomyopathy, atrial fibrillation with rapid ventricular response, and cardiac dysfunction. CT indicated a giant retroperitoneal mass measuring 33 cm in maximum diameter, with compression of the inferior vena cava. The pathological diagnosis was dedifferentiated liposarcoma. Preoperative interventional vascular embolization of the tumor was performed.

Anesthesia induction was achieved using etomidate, sufentanil, and rocuronium administered in small, divided doses to minimize hemodynamic depression during induction. Invasive arterial pressure and central venous pressure monitoring were established. GDFT was guided by cardiac output and stroke volume variation measurements obtained via the FloTrac device.

Following induction, in the setting of preexisting cardiac dysfunction and significant compression of the inferior vena cava, rapid atrial fibrillation with severe hypotension developed. Heart rate increased to a peak of 190 beats per minute, and invasive arterial pressure decreased to 70/40 mmHg. Continuous infusion of epinephrine was initiated to maintain arterial pressure. Amiodarone was administered for rate control (150 mg intravenously administered slowly, followed by continuous infusion at 20 mg/h), resulting in stabilization of the heart rate between 100 and 120 beats per minute. Concurrent moderate volume resuscitation was performed, leading to gradual hemodynamic recovery.

Intraoperative blood loss was 1,000 mL, and 800 mL of packed RBCs was transfused. The operative duration was 440 minutes. The resected tumor weighed approximately 6 kg. Postoperatively, the patient remained intubated and was transferred to the ICU. Extubation was performed 18 hours postoperatively, and the ICU stay lasted 5 days. Postoperative deep vein thrombosis developed in the lower extremities and resolved following anticoagulation therapy, with subsequent recanalization and organization of the thrombus. The patient was discharged on postoperative day 35.

## 4 Discussion

### 4.1 Anesthetic challenges in the resection of giant retroperitoneal tumors

Anesthetic management during resection of giant retroperitoneal tumors is associated with multiple challenges, among which hemodynamic instability represents the most critical concern. Large retroperitoneal masses may compress the inferior vena cava, resulting in reduced venous return and decreased cardiac preload. During anesthetic induction, these effects may be exacerbated by the vasodilatory and myocardial depressant properties of anesthetic agents, thereby increasing the risk of severe hypotension (6).

In this study, hypotension occurred immediately after induction in several patients and required prompt administration of vasoactive agents and volume resuscitation. Giant retroperitoneal tumors frequently adhere to or invade adjacent major vascular structures, including the abdominal aorta, inferior vena cava, and their branches. These vessels may be injured during surgical dissection and resection, resulting in massive hemorrhage (4).

In this cohort, 80% of patients experienced massive hemorrhage (≥ 1,000 mL), and 30% experienced severe hemorrhage (≥ 5,000 mL). The median blood loss was 2700 mL, with a maximum of 7,500 mL. Eighty percent of patients required blood transfusion, highlighting the substantial bleeding risk associated with these procedures. Reperfusion following tumor resection may precipitate significant hemodynamic fluctuations (7).

Close monitoring of circulatory fluctuations prior to and following tumor resection is essential, and vasoactive agents should be adjusted promptly as clinically indicated. In several patients in this study, blood pressure instability occurred following tumor removal and was effectively controlled through continuous hemodynamic monitoring and intraoperative abdominal compression (e.g., placement of a 1,000-mL saline bag) prior to complete tumor resection (8).

Respiratory system involvement warrants careful consideration. Giant masses may elevate the diaphragm, impair pulmonary function, and increase the risk of postoperative complications such as atelectasis or pneumonia (9). Preoperatively, 5 patients (50%) demonstrated moderate-to-severe restrictive ventilatory dysfunction or were unable to complete pulmonary function testing because of tumor compression. Six patients (60%) presented with preexisting hypoxemia (PaO_2_ < 80 mmHg), indicating substantial respiratory compromise attributable to compression by the giant retroperitoneal masses and diaphragmatic elevation. Postoperatively, bilateral pleural effusion and bilateral lower lobe atelectasis were observed in 2 patients (20%). These findings support the implementation of protective ventilation strategies during surgery in patients with preexisting respiratory impairment secondary to tumor compression. Prolonged postoperative respiratory support and intensified anti-infection therapy may be considered (10). Meanwhile, we found that TAP block alone could not provide sufficient postoperative analgesia for giant tumor resection, which may lead to delayed or inadequate recovery of respiratory function. In our cohort, 70% of patients required delayed extubation. Most of these decisions were planned in advance due to poor preoperative respiratory reserve and intraoperative hemodynamic instability. However, inadequate analgesia, elevated diaphragm and incomplete recovery of respiratory function may also have contributed. This suggests that more intensive multimodal analgesic strategies, such as continuous epidural analgesia or thoracic paravertebral block, may be needed. Whether the occurrence of postoperative pulmonary complications is related to inadequate analgesia warrants further investigation through larger-scale, multicenter, prospective studies, which is also our future research direction.

The prolonged operative duration and extensive surgical involvement further increased the complexity of perioperative management. In this study, the median operative duration was 260 minutes, with a maximum of 440 minutes. These factors necessitated meticulous fluid management, strict temperature regulation, and continuous monitoring of coagulation status.

### 4.2 Perioperative management strategies

From the experience derived from this study, several management strategies were beneficial in improving perioperative outcomes: preoperative preparation constituted the foundation of successful perioperative management. MDT consultations typically involved specialists from anesthesiology, surgery, radiology, interventional radiology, and the ICU. Through collaborative risk assessment and development of surgical and anesthetic plans, individualized perioperative management strategies were formulated, which played a critical role in optimizing clinical outcomes (11). In this study, 8 of the 10 patients (80%) underwent preoperative MDT consultation, providing important support for surgical management. 2 patients did not receive MDT management, primarily due to insufficient experience in handling early cases and the fact that a routine MDT process had not yet been established.

Preoperative interventional vascular embolization of the tumors or placement of occlusion balloons in major vessels was particularly important for tumors with abundant vascular supply, as these measures may substantially reduce the risk of intraoperative hemorrhage (12). In this cohort, 7 patients (70%) underwent preoperative vascular embolization, and 1 patient underwent placement of occlusion balloons in the abdominal aorta and iliac arteries; favorable intraoperative outcomes were observed in both contexts. Preoperative placement of a ureteral D-J stent represented an important protective measure to reduce the risk of ureteral injury during resection of retroperitoneal masses (13).

With respect to the selection of anesthetic technique, general anesthesia with endotracheal intubation remains the standard approach, and advanced hemodynamic monitoring is essential for optimal perioperative management (14). In this study, invasive arterial pressure monitoring was implemented in 90% of patients, central venous pressure monitoring in 80%, and advanced hemodynamic monitoring systems, such as FloTrac, were used in 60%, thereby facilitating more precise hemodynamic assessment and management. The failure to use FloTrac monitoring in 40% of cases was mainly due to limited availability of equipment and differences in individual anesthesiologists’ preferences. Nevertheless, we still observed the potential value of advanced monitoring for precise hemodynamic management.

Given the potential for rapid intraoperative hemorrhage and the need for prompt blood transfusion and fluid resuscitation, establishment of adequate intravenous access is critical. All patients who experienced massive hemorrhage were managed according to a massive transfusion protocol. Between 2 and 4 intravenous access routes (median, 3) were established to permit simultaneous administration of blood products, fluids, and high-dose vasoactive agents.

Fluid management should be guided by GDFT principles, based on hemodynamic monitoring parameters, including invasive arterial pressure, central venous pressure, cardiac output, stroke volume variation, and urine output. Infusion rates and fluid types should be adjusted accordingly to optimize volume status, enhance cardiac output, and maintain adequate oxygen delivery to vital organs. Prior studies have demonstrated that implementation of GDFT during tumor resection in adults can significantly improve tissue perfusion and clinical outcomes (15, 16).

For patients in whom major blood loss is anticipated, adequate blood products should be prepared in advance. During massive transfusion, coagulation function should be closely monitored, with timely supplementation of plasma, platelets, cryoprecipitate, fibrinogen, and prothrombin complex concentrate to reduce the risk of coagulopathy (17). In this study, one patient with hemorrhage reaching 7,500 mL received platelet transfusion in addition to fibrinogen and prothrombin complex concentrate. Another patient with a hemorrhage volume of 6,600 mL received fibrinogen and prothrombin complex concentrate. In these two patients, coagulation parameters showed marked changes during and after surgery. However, after aggressive supplementation with blood products, no clinically significant active oozing requiring interventional or surgical management occurred. According to the Expert Consensus on Perioperative Coagulation Management in Anesthesia (2020 Edition) (18), perioperative severe coagulopathy in this study was defined as follows: in the setting of massive hemorrhage, the presence of diffuse oozing that is difficult to control surgically, accompanied by any one of the following criteria: ① platelet count <50 × 10^9^/L, ② prothrombin time (PT) or activated partial thromboplastin time (APTT) >1.5 times the upper limit of normal, ③ international normalized ratio (INR) >2.0, or ④ fibrinogen <1.5 g/L, is considered to indicate the occurrence of perioperative severe coagulopathy. Therefore, we considered that these patients did not develop severe coagulopathy. Nevertheless, it must be acknowledged that massive transfusion inevitably carries some degree of dilutional coagulopathy. Due to the retrospective nature of this study, detailed dynamic coagulation profiles were not available, which is indeed one of the limitations of this study. Administration of vasoactive agents represents a central strategy for maintaining hemodynamic stability. In this study, vasoactive agents were administered in 100% of patients, primarily during episodes of hypotension following anesthetic induction, during hemodynamic fluctuations associated with tumor manipulation or resection, and after volume resuscitation when sustained blood pressure support was required.

### 4.3 Comparison with existing literature

When compared to previous studies on retroperitoneal tumors, the management approach applied in this study was largely consistent with existing reports; however, several distinguishing features were identified. Although prior literature has emphasized the value of advanced hemodynamic monitoring, its clinical importance was further supported by the findings of this study.

Notable aspects include the following: (1) the role of preoperative MDT consultation and preoperative vascular embolization was highlighted; these interventions have been less frequently detailed in the current literature; and (2) hemodynamic changes at critical intraoperative time points, along with corresponding management strategies, were described in detail, thereby providing practical guidance for clinical management.

### 4.4 Clinical significance and recommendations

From the findings and clinical experience derived from this study, several recommendations for anesthetic management are proposed. These measures may help improve perioperative outcomes. In terms of preoperative management, when conditions permit, MDT consultation should be performed for patients with complex specialist conditions, multiple comorbidities, and anticipated high surgical difficulty, in order to develop an individualized perioperative management plan. For tumors characterized by abundant vascularity, preoperative embolization should be considered to reduce the risk of intraoperative hemorrhage. Comprehensive preoperative evaluation, including imaging assessment with CT, magnetic resonance imaging, and related modalities, is essential to delineate the relationship between the tumor and major vascular structures and to assess operative risk. Adequate blood products should be prepared in advance, and reliable access for rapid transfusion should be established.

With respect to intraoperative management, invasive arterial pressure monitoring should be considered for all patients. Central venous pressure monitoring should be considered in patients at risk for significant blood loss or hemodynamic instability, and advanced monitoring modalities, such as FloTrac, are advisable in patients with complex clinical conditions. GDFT principles should be implemented, with fluid administration adjusted according to hemodynamic monitoring parameters. A massive transfusion protocol should be activated when indicated, and coagulation function should be assessed in a timely manner to reduce the risk of coagulopathy. In this study, 3 patients experienced intraoperative blood loss of ≥5,000 mL. To prevent dilutional coagulopathy, we supplemented platelets, fibrinogen and prothrombin complex concentrate during intraoperative transfusion of red blood cells and plasma, and continued intermittent postoperative infusion of red blood cells, fresh frozen plasma, fibrinogen and prothrombin complex concentrate. None of these 3 patients with massive hemorrhage developed clinically significant active oozing requiring interventional or surgical management. Therefore, we believe that aggressive supplementation of blood products, fibrinogen, and prothrombin complex concentrate after massive hemorrhage and massive transfusion helps prevent the development of dilutional coagulopathy. When available, thromboelastography (TEG) may facilitate more targeted correction of perioperative coagulopathy. Vasoactive agents should be readily available to enable prompt management of hemodynamic instability. Particular attention should be directed toward hemodynamic fluctuations during critical intraoperative phases, including anesthetic induction, tumor manipulation, tumor resection, and reperfusion.

With respect to postoperative management, patients at elevated risk, such as those who experienced massive hemorrhage, hemodynamic instability, or prolonged operative duration, should be considered for admission to the ICU. Close monitoring of vital signs, bleeding status, and respiratory function is essential to facilitate early identification of complications. Identified complications should be managed promptly to prevent further clinical deterioration.

### 4.5 Limitations of the study

This study has several limitations. Retrospective study designs are inherently susceptible to incomplete data and selection bias. In particular, the exclusion of emergency patients and those with ASA physical status ≥ IV meant that the zero mortality rate could not be extrapolated to higher-risk populations. Although rigorous data collection and quality control measures were implemented, the possibility of omissions or inaccuracies cannot be excluded. Retrospective designs do not allow adequate control of confounding variables, thereby limiting the ability to establish causal relationships. Only 10 patients were included, resulting in a relatively small sample size and limited statistical power. Multivariate analysis was not feasible, and independent prognostic factors could not be determined due to the small cohort. The limited sample size restricts the generalizability of the findings. However, given that giant retroperitoneal tumors represent a rare clinical condition and that detailed perioperative management data and experiential insights were provided, the findings may offer clinically relevant reference data in this challenging field.

As a single-center study, the results reflect institutional management practices. Variations in management protocols, technical expertise, and resource availability across institutions may influence outcomes; therefore, the findings may not be fully generalizable to other centers. Prospective multicenter studies with larger sample sizes are required to further validate and expand upon these management strategies.

## 5 Conclusion

This retrospective study assessed perioperative anesthetic management data from 10 patients who underwent resection of giant retroperitoneal tumors. Resection of these tumors was associated with substantial anesthetic challenges, particularly hemodynamic instability and a high risk of massive hemorrhage. Advanced hemodynamic monitoring, including invasive arterial pressure and central venous pressure monitoring, was essential for timely identification and management of hemodynamic changes. Concurrent implementation of proactive fluid management strategies and adequate preparation of blood products was critical for mitigating hemorrhagic risk. Preoperative measures, including MDT consultation and interventional embolization may help improve prognosis.

Individualized anesthetic management is essential, as each patient presents distinct clinical characteristics that require tailored strategies based on specific conditions. Future research should involve large, prospective multicenter studies to validate and generalize these management approaches, with the aim of establishing standardized perioperative management protocols. Evaluation of long-term outcomes, including 1-, 3-, and 5-year survival rates, should be incorporated into future investigations.

## Data Availability

The original contributions presented in the study are included in the article/supplementary material. Further inquiries can be directed to the corresponding author.
